# Temperature-controlled molecular switches in mammalian cells

**DOI:** 10.1016/j.jbc.2024.107865

**Published:** 2024-10-05

**Authors:** Eva Absmeier, Florian Heyd

**Affiliations:** 1 Laboratory of mRNA translation and turnover, Institute of Chemistry and Biochemistry, Freie Universität Berlin, Berlin, Germany; 2 Laboratory of RNA Biochemistry, Institute of Chemistry and Biochemistry, Freie Universität Berlin, Berlin, Germany

**Keywords:** molecular switches, temperature sensor, thermosensor, body temperature, heat shock, cold shock, circadian clock

## Abstract

Temperature is an omnipresent factor impacting on many aspects of life. In bacteria and ectothermic eukaryotes, various thermosensors and temperature-controlled switches have been described, ranging from RNA thermometers controlling the heat shock response in prokaryotes to temperature-dependent sex determination in reptiles, likely controlled through protein phosphorylation. However, the impact of subtle changes of human core body temperature are only beginning to be acknowledged. In this review, we will discuss thermosensing mechanisms and their functional implications with a focus on mammalian cells, also in the context of disease conditions. We will point out open questions and possible future directions for this emerging research field, which, in addition to molecular-mechanistic insights, holds the potential for the development of new therapeutic approaches.

## Temperature variations in mammalian cells

Temperature impacts all aspects of life and although mammalian body temperature is considered to be rather stable, quite some variations occur. In a healthy human body for example, core body temperature can vary up to 2 °C during the circadian cycle (reviewed in (1)). In addition, temperature ranges from 37 °C in the human body core to about 30 °C in the extremities, where the precise temperature is also dependent on the ambient temperature (reviewed in (2)). Even within a cell, there seems to be a temperature gradient, with potentially higher temperatures close to mitochondria (3). Body temperature also increases during exercise, fever, and potentially also due to enhanced environmental temperature, which is an increasing risk due to global warming and ensuing heat waves. On the other hand, upon cold exposure, core body temperature can decrease below 35 °C, a state called hypothermia, which is, in a controlled manner, used for its neuroprotective effect in some clinical settings (reviewed in (4)).

In general, mammalian cells are exposed to smaller temperature changes than for example plants, bacteria, or other ectotherms. Therefore, molecular temperature switches, dimmers, and sensors have to be extremely sensitive, as they react to comparably subtle temperature differences. Such mammalian temperature sensors are less well described than for example bacterial molecular thermometers. In this review we will briefly introduce temperature as a fundamental variable in biochemical reactions, define temperature switches and sensors, and present examples of temperature-controlled switches across different species. We will then summarize known examples in mammalian cells and give an outlook for this newly developing research field.

## Theoretical considerations and thermo-switches

Higher temperature goes along with higher reaction rates. As a general rule biochemical reaction speed increases 2- to 3-fold upon a 10 °C temperature increase. However, this linear behavior can be changed in biological systems by temperature sensitization. A high temperature sensitivity results in a large response to small temperature changes and *vice versa* for a small temperature sensitivity, which can be described by the Q10 value. Q10 is a dimensionless variable that describes the ratio of reaction rates measured at different temperatures (10 degrees apart) (5). Reactions with low Q10 values (*e.g.* below 4) are considered to be not very temperature sensitive, so that temperature changes act rather like a dimmer, whereas processes with high Q10 values (*e.g.* above 7) are considered to be highly temperature sensitive, and thus can act as molecular switches. Extreme examples are the activation kinetics of transient receptor potential (TRP) channels, which happens in a very narrow temperature range with a Q10 above 15 (see below for details). On the other hand, the circadian clock has evolved an elaborate system to be temperature independent (*e.g.* (6)). This temperature compensation is required to prevent a faster clock at higher temperature and leads to a Q10 of around 1 (= temperature insensitive) in a temperature range between 34 °C and 40 °C. While in these examples, enzymatic activity or the properties of ion channels are altered, temperature switches do not always need to affect enzymes, as for example RNA structures can serve a similar purpose. The network of non-covalent bonds, which proteins and RNA form, are highly sensitive to temperature changes. These interactions include electrostatic-, hydrophobic- and pi-interactions, hydrogen bonds and van der Waals forces, which typically release 1 to 5 kcal/mol upon the formation of the interaction. Hydrogen bonds, which are especially important for RNA secondary and tertiary structures, vary in strength between 2 to 7 kcal/mol, depending on the precise atoms involved. These above-mentioned strengths are for non-covalent interactions in isolation, but their strength is usually lower in the context of a protein (0.5–1.5 kcal/mol (7)), or an RNA in solution. For example, the free energies of various RNA stem loops are calculated to be within a range of −3 to −6 kcal/mol (8) and reviewed in (9, 10). Given such low free energy it is well conceivable that even a 1 °C change in temperature, as is frequently observed in the human body, can influence the stability of RNA and protein structures thereby impacting on all functional aspects of a cell. This may happen in a switch-like manner, but could also work like a dimmer and shift the equilibrium between alternative secondary structures to favor one of two or several functional states.

## Temperature sensors in non-mammalian species

To date, only a few examples of temperature-controlled molecular switches in mammalian cells have been described. We will start by briefly highlighting some temperature switches in other organisms to show how variable these switches can be and that they impact on a wide variety of cellular mechanisms (reviewed in (5)) and then describe known temperature switches in mammals.

### Bacteria

**Figure 1:**
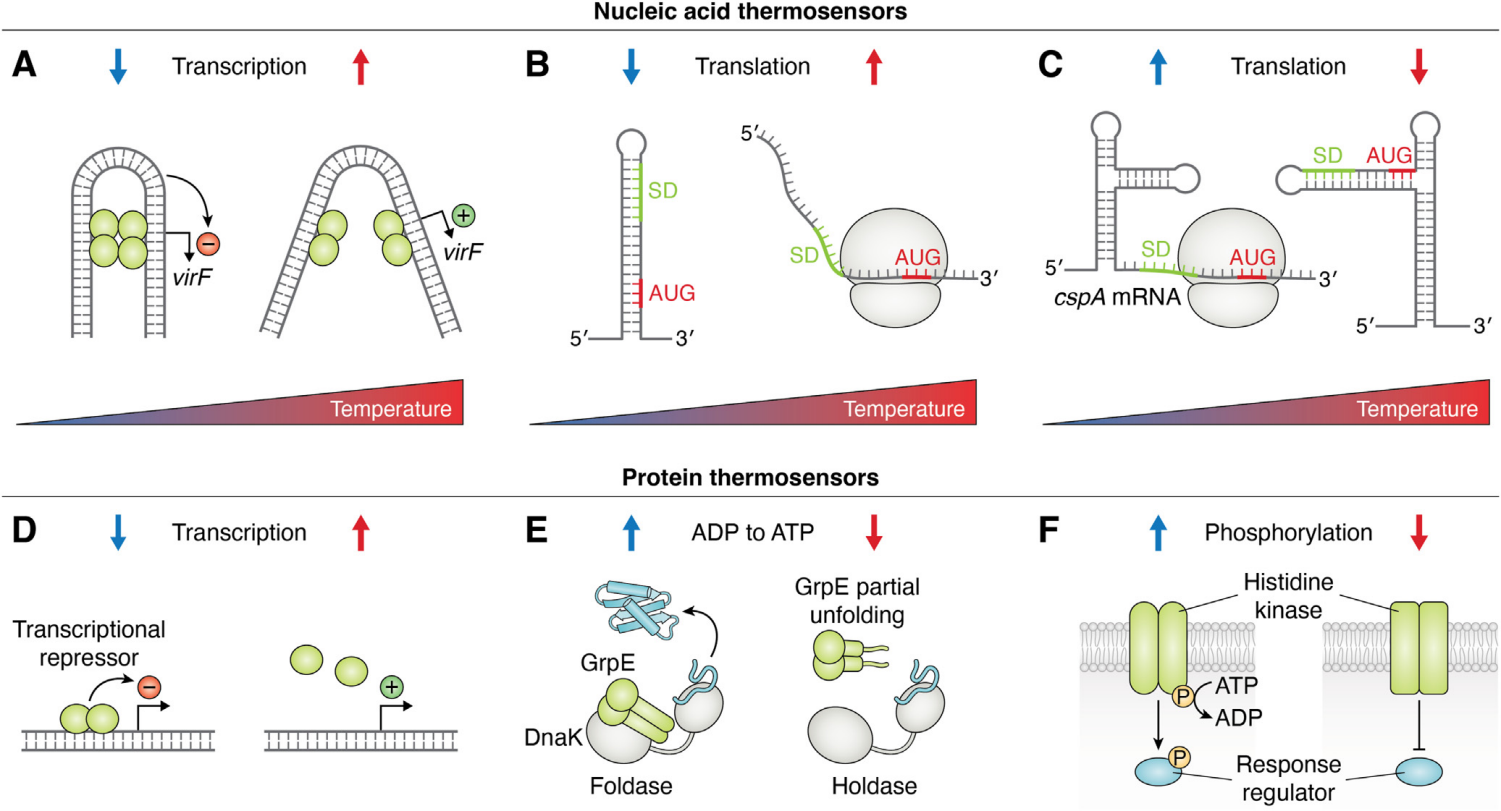
**Examples of bacterial thermosensors.** Top: Nucleic acid thermosensors. *A*, DNA-temperature sensor: The virF promotor is occluded by a DNA bend at low temperatures, allowing binding of repressive proteins (*green*). The bend melts at higher temperatures and allows transcription. *B*, RNA-temperature sensor: Secondary structures in the 5′untranslated region (UTR) of a messenger RNA (mRNA) inhibit translation at low temperatures, by occluding the Shine-Dalgarno sequence (SD) and the start codon (AUG) (*red*). Melting of the secondary structures at higher temperatures allows translation of the mRNAs (bound ribosome). *C*, RNA-temperature sensor: The cspA mRNA adopts different secondary structures at low *versus* high temperatures. The secondary structures at high temperatures occlude the SD sequence and the start codon (*red*), inhibiting translation, whereas the secondary structures at low temperatures allow translation. Bottom: Protein-temperature sensors. *D*, repressive proteins (*green*) dimerize at low temperatures and repress transcription by DNA binding. This repression is alleviated at higher temperatures. *E*, the nucleotide exchange factor GrpE (*green*) binds to the DnaK chaperone (*grey*). Higher temperature leads to partial unfolding of GrpE, leading to slower ADP to ATP exchange and rendering DnaK to a holdase instead of a foldase of the bound unfolded protein. *F*, Histidine-kinase transmembrane receptors act as temperature sensors by auto-phosphorylation and subsequent signal molecule phosphorylation at low temperatures. Signaling is inactive at higher temperatures. Figure is partially adapted from (5).

Bacteria evolved a plethora of nucleic acid-based temperature sensors (Fig. 1, top). An example for a DNA-based temperature sensor is found in the infectious *Shigella* bacterium, where expression of a virulence factor for host invasion reacts to the host body temperature. Expression of virF is repressed at lower temperatures by occlusion of the promotor by a DNA bend, induced by repressive proteins (Fig. 1*A*). This bend relaxes at temperatures above 32 °C and transcription is initiated (11, 12). Similarly, a variety of RNA-thermometers are present in bacteria (Fig. 1, *B* and *C*). In many cases, they form secondary structures, up to four hairpins, in the 5′untranslated region (5′UTR) of a messenger RNA (mRNA), occluding the Shine Dalgarno sequence and thereby inhibiting translation of, for example, virulence factors and heat shock proteins at lower temperature (reviewed in (13, 14)). Increased temperature then melts these secondary structures and allows translation of the respective mRNAs (Fig. 1*B*). An example of such an RNA thermosensor is the 5′UTR of the rpoH mRNA, which encodes for the alternative RNA polymerase σ-factor, σ^32^ (reviewed in (13, 14)). This often happens within a narrow temperature range, *e.g.* between 37 °C and 42 °C where the bacterial heat shock response is initiated, and can be controlled in a switch-like manner or like a molecular dimmer (reviewed in (13)). On the other hand, RNA secondary structures also control the cold shock response in bacteria, again through alternative RNA conformations that alter accessibility of the translation start site of, for example, the cspA mRNA (15) (Fig. 1*C*). Bacteria also employ protein thermosensors, examples are the TlpA protein in *Salmonella* (16), RovA in *Yersinia* (17) and RheA in *Streptomyces* (18), which change their conformation and DNA binding activity due to temperature changes, resulting in altered transcription (Fig. 1*D*). Temperature can also influence proteins involved in protein quality control. An example is the bacterial DnaK chaperone system, where the GrpE co-chaperone partially unfolds at temperatures above 40 °C and thus losing its nucleotide exchange activity. This leads to reduced ADP dissociation from DnaK, which remains in the high affinity state, not releasing its protein substrate (holdase) (Fig. 1*E*) (19). Other examples of protein thermosensors in bacteria include the histidine kinase/response regulator signaling module (Fig. 1*F*). In this system a membrane bound sensor reacts to changes in temperature by autophosphorylation of a histidine residue in the intracellular protein domain. The phosphate group is then transferred to an intracellular response protein (reviewed in (20)). Examples are the *Pseudomonas syringae* CorS/CorR system (21), the Agrobacterium tumefaciens VirA/VirG system (22) and the *Bacillus subtilis* DesK/DesR system (23). In the latter, the DesK histidine kinase detects different temperatures based on varying membrane thicknesses (24). There are more examples for thermosensors in bacteria, which are reviewed elsewhere (5, 25).

### Eukaryotes

Temperature switches, dimmers, and sensors similar to the ones found in bacteria are also found in eukaryotes, in particular in exothermic eukaryotes. In *Drosophila*, the impact of temperature has been studied in different developmental processes, in part in molecular detail. For example, *Drosophila* eggs can develop to adult flies in a temperature range between 12 °C and 32 °C, where ovariole numbers peak at 22 °C and wing lengths peaks at 16 °C for female flies (26). *Drosophila* brain development is also highly temperature dependent, with the formation of neuromuscular synapses being more efficient at higher rearing temperature, potentially controlled through altered local translation (27). The *Drosophila* heat shock response may show similarities to the induction of the bacterial heat shock response, as RNA secondary structures have been found in the 5′UTR of the *Drosophila* Hsp90 mRNA, which may act as a temperature-switch to allow translation only at warmer temperature (28). In yeast, the heat shock response is (at least in part) controlled through protein-based thermo switches that alter the formation of RNA-protein aggregates thereby allowing selective translation of mRNAs required for the heat shock response at higher temperatures. This response is activated in a very narrow temperature range and controlled through conformational switches in translation factors and RNA helicases (29, 30). For example, in yeast the helicase Ded1p (homolog of the mammalian DDX3), which facilitates translation through resolving secondary structures in 5′UTRs to allow ribosomal scanning, phase separates upon heat shock and forms aggregates also containing translationally silenced mRNAs. This leads to preferential translation of heat shock proteins, as the respective RNAs tend to have shorter and less structured 5′UTRs and are therefore less dependent on Ded1p. Interestingly, the temperature at which Ded1p phase separates is adapted to the living temperature of diverse fungi, further underlining the important role of this phase separation event in determining the temperature that different organisms consider as heat (29, 30). Preferential translation of heat shock proteins is further increased through condensation of components of the eIF4F complex, which contributes to the formation of heat shock granules and reduces translation of house keeping genes (29, 30). Another temperature sensing mechanism in yeast that is also dependent on the formation of a larger protein assembly relies in the small heat shock protein Hsp26. Hsp26 forms large oligomeric structures, which dissociate only at elevated temperatures, which then allows the dimeric form of Hsp26 to bind to unfolded proteins and to act as chaperone (31). While these mechanisms have been mainly investigated in yeast, the concept of temperature-controlled phase separation seems well-suited to also control the response to temperature changes in mammals.

The circadian clock is another example where several temperature-based mechanisms are at work in eukaryotes. In *Drosophila* for example, the 3′UTR of the period pre-mRNA contains an intron which is more efficiently spliced at lower temperatures, which then controls the expression of the PER protein and the circadian clock (32). In the fungi *Neurospora crassa* the circadian clock protein *frequency* is expressed in a temperature-dependent manner due to increased translation initiation at non-consensus Kozak sequences upstream of the *frq* circadian gene at low temperatures (33).

A very fundamental temperature-controlled switch is observed in some reptiles, where the temperature at which the eggs are incubated during a specific developmental period determines the sex of the offspring in a process called temperature-dependent sex determination (TSD) (reviewed in (34)). TSD has been known and studied for decades, but the molecular temperature sensor that translates subtle differences in temperature into female or male developmental programs remains unknown. Recent evidence suggests the involvement of phosphorylation events in STAT3, which then controls transcription of the epigenetic regulator Kdm6b (35), but the underlying temperature-dependent kinase has not yet been identified.

Another interesting temperature switch has recently been discovered in octopus. Here, temperature-dependent RNA editing and recoding lead to differential expression of diverse proteins according to the water temperature. For example, neurotransmitter release and microtubule-dependent transport are altered in different temperatures, allowing rapid adaptation to changing environmental conditions (36, 37). These examples show how diverse temperature-controlled switches are in terms of the molecular mechanism and the ensuing functionality. It is also interesting to note that many temperature switches react to subtle temperature differentials, *for example,* TSD is controlled in a range of a few degrees Celsius. Such temperature differentials can easily occur in the human body as well, for example*,* during high fever, or in a tissue-dependent manner, with for example skin and testis being kept approximately 5 °C below core body temperature. However, most of the mechanisms described earlier have not been studied in human or other mammalian systems and therefore, the molecular and functional impact of subtle temperature changes in humans is only beginning to be acknowledged.

## Temperature sensors in mammalian cells

### Ion channels

**Figure 2:**
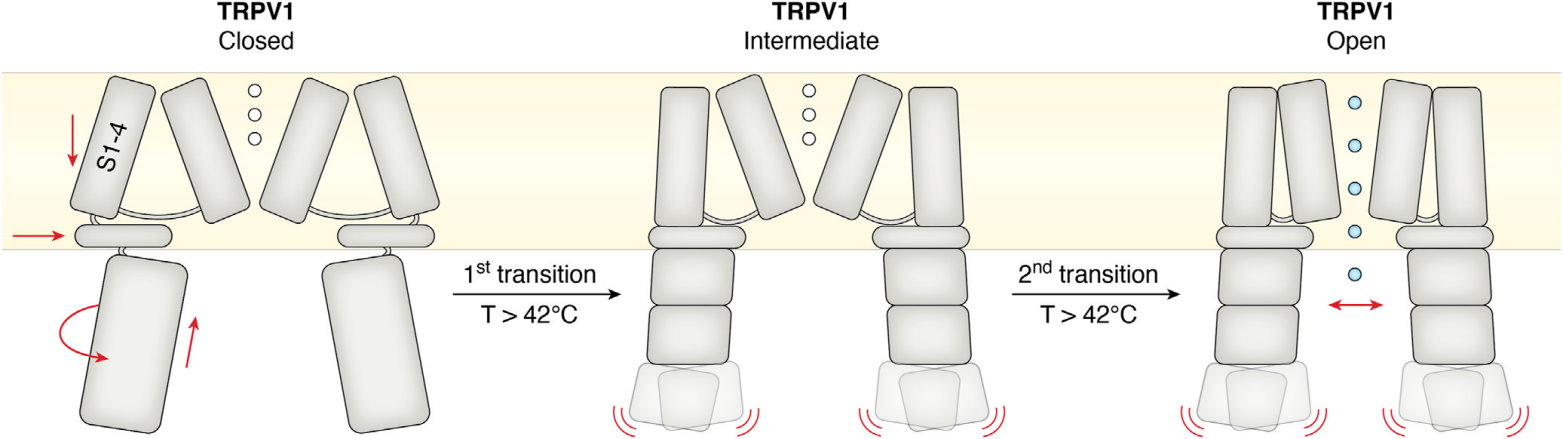
**Model of heat sensing by TRPV1.** The model is based on several cryoEM structures of recombinant *Rattus norvegicus* TRPV1 embedded in nanodiscs. For the cryoEM structures, all samples were bound to capsaicin and incubated at different temperatures before plunge-freezing. In the elevated temperature sample, two conformations were observed (intermediate and open), suggesting two temperature-dependent transitions. In the first transition, major rearrangements take place in the cytosolic and the transmembrane domains. In the second transition, local conformational changes open the pore. The figure is adapted from (41). Rearrangements of the transmembrane helices S1-4 upon temperature increase are in agreement with studies on the isolated S1-4 domain (42).

A well-known class of proteins that change their activity as a function of temperature changes are ion channels of the conserved TRP family. Different family members are sensitive to different temperature ranges, from noxious heat to innocuous warmth and cold to noxious cold. Individual primary sensory neurons express (redundant) receptors that react to the same temperature range (reviewed in (38)). Their activation triggers an action potential that is then perceived and processed in specialized areas in the brain where, depending on the temperature range that led to the activation, responses are triggered (reviewed in (39)). TRP channels have a very high Q10, in some cases above 20, meaning that they act extremely sensitive to changes in their particular temperature range (reviewed in (38)). This allows a switch-like behavior as these channels go from an essentially closed conformation to an open one within a very small temperature range. For example, TRPV1, a receptor that senses noxious heat and that is also activated by capsaicin (40), a substance that is a component of chili pepper, displays a Q10 above 15 and an activation threshold of 42 °C, which coincides with the temperature perceived as heat pain in humans. Deleting TRPV1 from the mouse genome does not completely abolish heat sensing, whereas this is the case when TRPV1 positive cells are deleted (reviewed in (39)). This suggests that additional heat-sensitive TRPs are coexpressed in TRPV1-positive cells, likely to create some redundancy for the perception of potentially life-threatening temperatures. From a molecular-mechanistic perspective, the high Q10 in a particular temperature range and the ensuing switch-like behavior are very interesting and, despite considerable effort, not fully understood. For TRPV1 cryoEM structures have revealed different conformations for example depending on the binding of the activator capsaicin (Fig. 2) (41). These structures together with chimeric and mutant receptors suggest that temperature sensitivity is mediated by the transmembrane domain of the receptor, but molecular and/or atomic details how the high Q10 in a specific and narrow temperature region is achieved are only beginning to be understood. Recent work has suggested the involvement of some key amino acids in the transmembrane domain of TRPV1 in thermosensing (42). This was followed by cryoEM structures that provide first evidence for temperature-controlled conformational changes as the basis for thermosensing (41, 43, 44), which will be very valuable to obtain a more detailed understanding of how the precise temperature range and the very high Q10 values are achieved. In addition to protein-mediated thermosensing, temperature-controlled changes in membrane fluidity and structure may contribute to increase the Q10 of TRP receptors. TRP receptors are mainly expressed in primary sensory neurons and are coupled directly to specialized regions in the brain responsible for temperature perception and integration. This system thus acts on the level of the whole organism and can create behavioral output as response to changing temperature.

### Heat shock proteins

**Figure 3:**
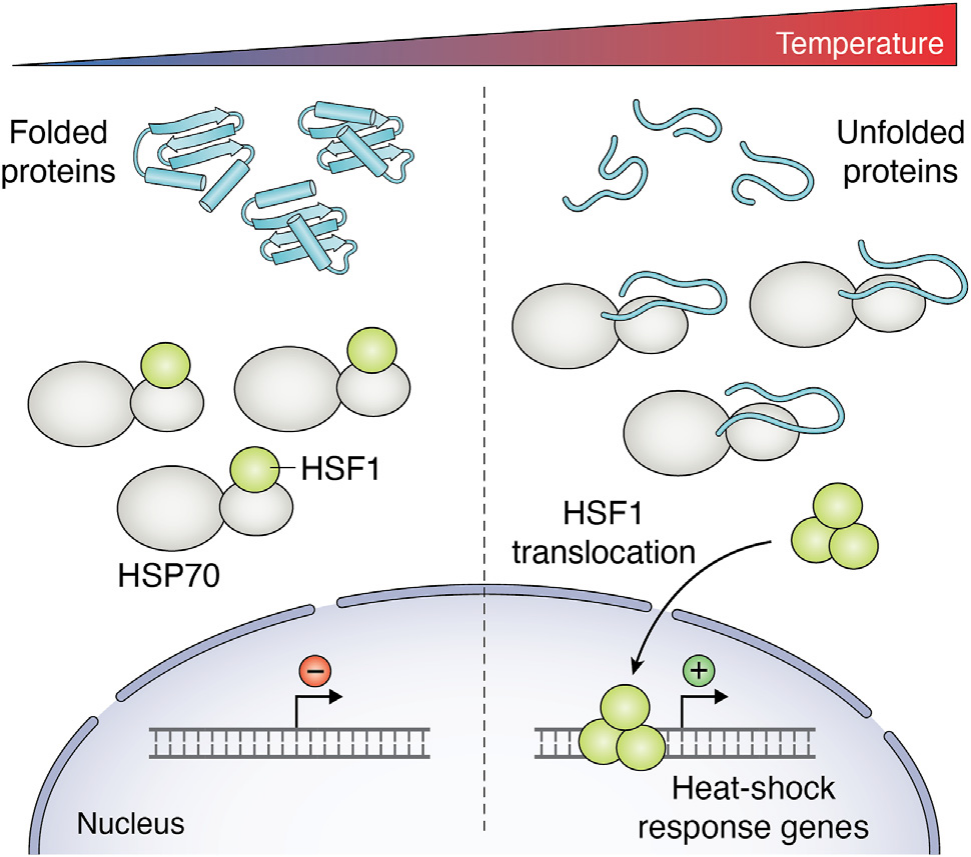
**HSP70 and HSF1 heat-shock response in mammals.** (*Left*) At low temperatures, most proteins are folded. HSF1 transcription factor is bound to HSP70, which keeps it in an inactive state. (*Right*) At high temperatures, more unfolded proteins are present, engaging with HSP70 and releasing HSF1. Trimeric HSF1 can then translocate to the nucleus and activate the transcription of heat-shock response target genes.

In contrast, heat shock and cold shock responses are cell-autonomous reactions designed to protect individual cells from potentially harmful temperatures (reviewed in (45)). The heat shock response in mammalian cells is activated at temperatures above 39 °C and typically reaches high activity at 42 °C. Given this narrow temperature range, it can be considered a temperature-dependent molecular switch. One consequence of unphysiologically high temperatures is the unfolding and misfolding of proteins. In addition to directly disturbing the function of individual proteins, this leads to exposure of hydrophobic protein parts, which, under native conditions, make up the protein core and are thus hidden from the aqueous environment and are not available for intermolecular interactions. Exposure to these hydrophobic regions can induce the formation of protein aggregates that quickly become toxic to the cell. One major task of the heat shock response is to increase the expression of protein chaperones that help to refold misfolded proteins into their native conformation and to prevent the formation of toxic protein aggregates (reviewed in (45)). One elegant way of inducing the heat shock response relies on sensing unfolded proteins by the heat shock protein 70 (HSP70) (Fig. 3). In the absence of unfolded proteins, HSP70 is bound to heat shock factor 1 (HSF1), the main transcription factor of the heat shock response. In this complex, HSP70 keeps HSF1 in an inactive conformation (Fig. 3, left). Once unfolded proteins are present, HSP70 binds to them, thereby releasing HSF1. This allows HSF1 to form the active trimers that can then bind to the promoter of target genes to initiate the transcriptional heat shock response (reviewed in (46)) (Fig. 3, right). As HSP70 binds to unfolded proteins in general, the release of HSF1 does not depend on one specific molecular event, but is rather dependent on global protein unfolding. Thermosensing in this system is therefore set to a temperature range that corresponds to the melting temperature of the global proteome, or at least to the temperature range that leads to unfolding of enough proteins to titrate HSP70 away from HSF1. Recent studies could show that HSF1 additionally acts as a thermosensor itself. Upon increase in temperature, a C-terminal region of HSF1 unfolds, resulting in enhanced trimerization by an N-terminal region and thus DNA binding. This thermosensing event is dependent on the concentration of HSF1 and can therefore be adapted to different cellular requirements (47, 48).

The same molecular system could therefore be used in organisms with different body temperatures and a different threshold temperature for heat shock activation, if the global melting temperature of the proteome is adapted to the respective temperature range of different organisms. Rather than specific changes in the temperature sensor, a global adaptation of the proteome’s melting temperature would determine what the cells of different organisms consider as hot. In mammals, heat shock is closely related to fever, an evolutionarily younger response that is essential to fight pathogens (reviewed in (49)). Both pathways overlap in part, as the body temperature reached during fever activates the heat shock response and accordingly, HSF1 is also involved in controlling the expression of some cytokines involved in the immune response (reviewed in (50, 51)). Interestingly, in addition to the above-described cell-autonomous response, in *Caenorhabditis elegans*, a non-cell-autonomous heat shock regulation was identified. This response is achieved through thermosensory neurons and was suggested to coordinate the heat shock response in individual cells across different tissues, which may be a mechanism present in other multicellular organisms as well (52).

### Body temperature changes in the normothermic range

In physiological settings in humans, small temperature differentials in the normothermic range occur in various conditions. A prominent example is reduced core body temperature in older individuals, which may act as a systemic signal that alters cellular homeostasis. One example is the observation that a specific subunit of the proteasome, PSME3, is induced at 36 °C. This has been suggested to reduce the formation of neurotoxic protein aggregates and contributes to cold-induced longevity in *C.elegans* (53). Reduced body temperature upon aging may thus be used to counteract accumulating neuronal damage and neurodegenerative diseases, which typically affect older individuals. On the other hand, a recent study showed that lower body temperature decreases the magnitude of the innate antiviral immune response. This is mediated by temperature-controlled processing of the STAT2 pre-mRNA, which leads to higher STAT2 levels at higher temperatures in a switch-like manner, as the increase happens specifically between 37 °C and 38 °C. Higher STAT2 expression then increases the ability to mount an antiviral immune response, which may contribute to higher susceptibility of older individuals with lower body temperature for severe SARS-CoV-2 infections when compared to children with higher body temperature (54). These examples underline the fundamental impact of small changes in body temperature on essential functionality. Another important point is that changes in body temperature impact very diverse physiological processes and temperature changes leading to a favorable outcome in one process may have an adverse effect on others.

### Cold-shock proteins

As the heat shock response, the cold shock response in mammals is initiated by a temperature differential of approximately 2 °C, starting below 35 °C. However, functional consequences of cold shock such as a global inhibition of Cap-dependent translation and increased IRES (internal ribosomal entry site) -mediated translation require lower temperatures and show a gradual behavior, more like a molecular dimmer than a switch (reviewed in (55)). An interesting analogy between heat shock and cold shock is that heat shock favors the aggregation of proteins, whereas colder temperatures increase the propensity of RNAs to form aggregates. Consistently, the two most prominent cold shock proteins in mammals, RBM3 (RNA binding motif 3) and CIRBP (cold-induced RNA binding protein) are both RNA binding proteins that have been suggested to act as RNA chaperones (reviewed in (56, 57)). Although their precise molecular function remains to be described, it is tempting to speculate, that just like protein chaperones during heat shock, CIRBP and RBM3 act to prevent unwanted formation of RNA aggregates upon cold exposure. This idea has been put forward and an analogy between misfolded protein aggregation and RNA-dependent stress granules has been suggested, that are counteracted by heat shock proteins/protein chaperones or RNA chaperones, respectively (reviewed in (58)). It should be noted that the temperature-dependent expression of RBM3 and CIRBP follows a different mechanism than the heat shock response. Cold induction of RBM3 and CIRBP appears to be independent of global RNA aggregation but is rather controlled by specific, temperature-sensitive processing of the CIRBP and RBM3 pre-mRNAs that will be discussed in more detail below. However, whether there are mechanisms, analogous to the heat shock response, that globally sense aberrant RNA aggregation at colder temperatures to then induce RNA chaperones remains an open question.

### Kinase activity and alternative splicing as temperature-controlled molecular switches

**Figure 4:**
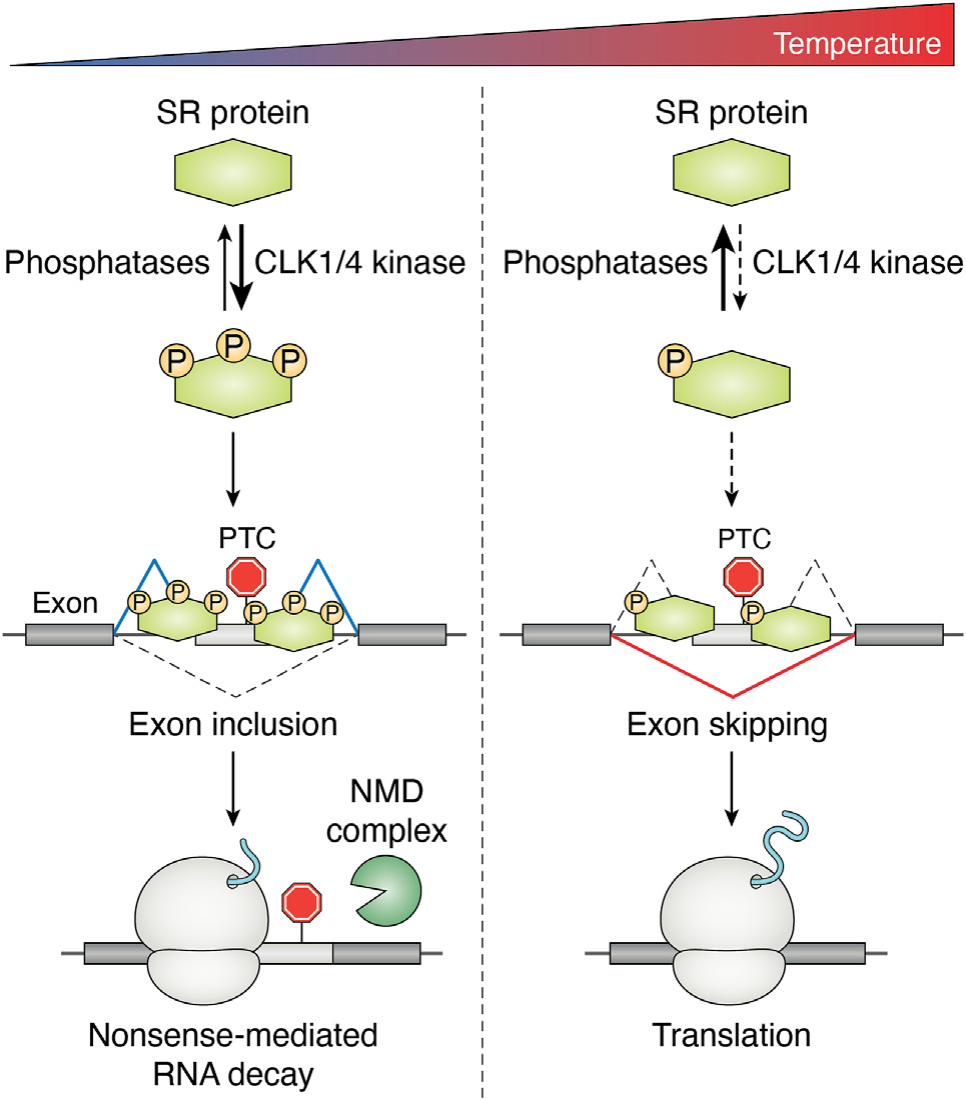
**Alternative-splicing coupled to nonsense-mediated decay.** (*Left*) CLK1/4 kinases are more active at low temperatures, which increases the phosphorylation of SR proteins. This, in turn, controls alternative splicing, in the example shown here it leads to the inclusion of an exon containing a premature termination codon (PTC, Stop sign), resulting in the activation of the nonsense-mediated RNA decay pathway. (*Right*) At higher temperatures, CLK1/4 is less active and SR protein phosphorylation is reduced. This leads to the exclusion of an exon containing a PTC, resulting in the translation of the full-length protein and normal translation termination. This is only one example of how temperature-dependent SR protein phosphorylation can control alternative splicing and other mechanisms are possible.

CIRBP and RBM3 have long been known as the most prominent cold shock proteins in mammals. Their induction happens gradually, but given the narrow temperature range of their induction, *i.e.* between 38 °C and 34 °C, appears switch-like. Recent work has shown that this induction is not controlled through changes in *de novo* transcription, but rather through temperature-regulated mRNA stability. More specifically, changes in the body temperature range cause a switch in alternative splicing of exons containing premature termination codons (PTCs), which leads to degradation of the transcripts by the nonsense-mediated decay (NMD) pathway (Fig. 4). This process together is called alternative-splicing coupled to nonsense-mediated decay (AS-NMD) (reviewed in (59)), which plays a major role in determining the temperature-dependent transcriptome as will be discussed below. We have recently shown that the subtle circadian changes in body temperature are sufficient to globally control alternative splicing and that this concerted splicing switch is temperature-driven and independent of the core circadian clock work (60, 61). The thermosensors that connect subtle temperature changes with splicing regulation are a family of broadly expressed kinases, Cdc2-like kinases (CLKs) (reviewed in (62)). CLK1 and CLK4 are ubiquitously expressed and react to changes in temperature in the physiologically relevant temperature range with a highly sensitive change in activity. Although a Q10 value was not formally calculated, human and mouse CLKs 1 and 4 are basically switched on and off between 35 °C and 38 °C, thus acting as temperature-controlled switches (63). It is noteworthy that these kinases display higher activity at lower temperature (Fig. 4), thus acting against the Q10 rule. This is enabled by a temperature-controlled conformational switch in the active center of the kinase, that likely blocks substrate access at higher temperature (63), but molecular and atomic details that allow such sensitive thermosensing remain to be elucidated. CLKs as thermosensors are evolutionarily conserved and adapted to the living temperature of diverse organisms in a way that they are switched off at the upper end of the physiologically relevant temperature range. It should be noted that another family of kinases, p38, has also been suggested to undergo conformational changes in the physiologically relevant temperature range (64), suggesting that thermosensing coupled to phosphorylation cascades is a more general phenomenon.

The main substrate of CLKs is serine and arginine-rich proteins (SR proteins), a family of RNA-binding proteins (RBPs) that are known to control every aspect of RNA processing, from splicing to nuclear export, translation, and degradation (reviewed in (65)). Importantly, the activity of SR proteins is controlled through their phosphorylation status, which is used to control alternative splicing but also splicing in general, for example, dephosphorylated SRSF10 globally represses splicing during heat shock and during mitosis (66, 67). The phosphorylation status of SR proteins is controlled through CLKs and another family of kinases, SRPKs (SR protein kinases), and phosphatases, which are less well-defined. SRPK activity is remarkably stable over a large temperature range (63), leaving CLKs as thermosensors that directly couple changes in temperature with SR protein phosphorylation and RNA processing. Altered SR protein phosphorylation at different body temperatures is instrumental in controlling alternative splicing, which was shown for individual target exons (61, 68) as well as globally by pharmacological manipulation of CLK activity at different temperatures. These studies initially focused on cassette exons whose alternative splicing removes or adds parts of the coding sequence and which can therefore impact on protein function without directly altering mRNA and protein abundance. Further analysis then addressed whether the same mechanism, through AS-NMD, can also control the abundance of mRNAs in a temperature-dependent manner. One of the first examples was the CIRBP mRNA, which is strongly cold-induced, but the mechanistic basis was not known until then. A detailed analysis of temperature-regulated CIRBP mRNA expression revealed the presence of an NMD isoform at higher temperatures, which was dependent on CLK activity. CRISPR/Cas9-mediated deletion of the NMD-inducing segment strongly reduced temperature sensitivity and led to high CIRBP expression already at high temperatures (63). Later on, the same mechanism was shown to be responsible for the temperature-regulated expression of the other cold shock protein, RBM3. For RBM3 a cassette exon containing several PTCs was shown to be heat-included and genome-engineered cell lines lacking this exon demonstrated that AS-NMD is solely responsible for switching on RBM3 expression at colder temperatures. Importantly, AS-NMD can be manipulated using splice-switching antisense oligonucleotides, which may offer a way of increasing expression of the broadly neuroprotective RBM3 at normothermia. This approach is currently being pursued to test its applicability as a new therapeutic approach in a wide range of neurodegenerative conditions, ranging from acute brain damage through hypoxia to chronic conditions such as age-related dementias (69).

Furthermore, the above-mentioned STAT2, which induces antiviral immunity at higher temperatures is also controlled through AS-NMD, in this case through a cold-induced NMD isoform (54) (Fig. 4), suggesting a more general role of AS-NMD as a temperature-controlled molecular switch. To gain global insight into the role of AS-NMD in temperature-controlled gene expression, the translation inhibitor cycloheximide, which also inhibits translation-coupled mRNA decay processes such as NMD, was used. RNA-Seq revealed many splicing isoforms that are temperature-controlled and only present in the presence of cycloheximide. These experiments suggest a major impact of AS-NMD on temperature-controlled gene expression, which is independent of regulated transcription (70). In addition to suggesting a mechanistic basis for temperature-controlled gene expression, this model provides an alternative mechanism that could contribute to rhythmic gene expression in endothermic organisms with circadian body temperature cycles. The oscillating expression of many genes could actually be controlled through temperature-controlled splicing switches that lead to NMD rather than through the classical core clock-driven time of the day-dependent changes in transcription. While the result in a healthy individual is the same, oscillating expression of many genes, the underlying systemic signal and molecular mechanisms are very different and the relative contribution of the two mechanisms in generating rhythmic gene expression remains to be determined. Such knowledge may become especially important in conditions with disturbed or reduced phase and amplitude of rhythmic gene expression, such as shift work or ageing, as targeted interventions and potential restoration of canonical oscillation requires knowledge about the underlying molecular mechanism.

### Temperature and the circadian clock

In addition to the potential role of CIRBP as an RNA chaperone, CIRBP has been shown to be involved in temperature-dependent synchronization of the circadian clock. Body temperature is under circadian control, with higher temperature during the active phase and lower body temperature during the inactive phase and sleep. Other variables such as the feeding status or physical exercise contribute to set the precise body temperature at a given time, but the mean body temperature follows a circadian pattern (reviewed in (1)). This is controlled through a specialized region in the brain, the suprachiasmatic nucleus (SCN), which contains the central clock, also called master clock. The SCN receives direct light input, which sets the central clock, and then uses different signals to synchronize peripheral tissues with one another and the environment (reviewed in (71)). One of the systemic signals that the SCN uses for the synchronization of peripheral tissues is circadian body temperature cycles. Importantly, the central clock in the SCN itself is not entrained by temperature, as this would not be compatible with the generation of oscillating body temperature by the SCN (72). The molecular basis that allows the central clock to resist entrainment by temperature requires a cellular network within the SCN, as preventing cell-cell communication in the SCN creates temperature-sensitive individual cells (72). However, the precise molecular mechanism that creates a temperature-insensitive clock in the SCN, whereas peripheral clocks use temperature as an entrainment signal, remains to be uncovered. In peripheral clocks, temperature entrainment is at least in part achieved through rhythmic expression of CIRBP, which, as expected, shows highest expression during the inactive phase with lower body temperature. CIRBP has been shown to bind to the CLOCK mRNA, which encodes for a core component of the molecular clock work and is required for robust oscillation (73). As CIRBP, RBM3 is also expressed in a rhythmic manner with higher expression during the inactive, colder phase. Together, temperature-controlled CIRBP and RBM3 expression have been suggested to control rhythmic changes in poly(A) site selection, which may contribute to establishing circadian functionality (74). On the other hand, HSF1 has been shown to be induced at the beginning of the active phase when body temperature increases (75) and might therefore also contribute to entrainment through temperature cycles. While the regulation of CIRBP and HSF1 has also been addressed *in vivo* in mice, the impact of temperature on the synchronization of the core clock can be recapitulated in cell culture systems. Here synchronization can be achieved by switching the temperature every 12 h, typically between 34 °C and 38 °C, which mimics body temperature cycles, although a larger amplitude than is observed *in vivo* is used in cell culture experiments.

## Outlook—RNA structures as thermosensors in human cells and potential therapeutic applications

As discussed above, RNA thermometers regulate the translation of diverse proteins during environmental temperature changes in bacteria and other organisms. It is intriguing to speculate that RNA structure-dependent translation switches also exist in mammals. Translation regulation could be achieved in various ways, such as by structures in the 5′ or 3′UTRs that could control translation initiation in the 5′UTR or binding of miRNAs to the 3′UTR. In addition, upstream ORFs (uORF) regulate translation by trapping the scanning initiation complex and thereby inhibiting translation of the main ORF. Recently, such a regulatory minimal uORF (a start-codon is directly followed by a stop codon) has been identified in the clock component PER2 mRNA (76). Surprisingly, this minimal uORF enhances Per2 protein translation in synchronized cells during the rising phase of Per2. Although the molecular details of how a minimal uORF can regulate translation are unknown, it is an interesting example of how untranslated regions can regulate translation, potentially through RNA structures that are controlled in a body temperature dependent manner. Another possibility is temperature-controlled alternative splicing that can affect untranslated regions. For example, we have recently shown that the 5′UTR of the Tata-box binding protein (TBP) is alternatively spliced in a body temperature-dependent manner in mice, thereby controlling translation efficiency and the amount of TBP. In this mechanism, it is not the RNA that adapts different conformations, but different RNAs that are produced by alternative splicing, that are predicted to form alternative secondary structures (61). Temperature-controlled RNA secondary structures could also be involved in directly regulating alternative splicing through controlling the accessibility of splice sites. An example are RNA G-quadruplexes that are enriched close to the splice sites of cassette exons (77) and could act as temperature-dependent switches to control alternative splicing of such exons. In plants, cold-induced formation of RNA G-quadruplexes in 3′UTRs has been suggested to control RNA stability (78), which could also act in controlling mammalian gene expression.

Temperature switches in mammals will not only be interesting from a basic science and mechanistic perspective but also have a large potential for translational applications. Thermotherapies are used in diverse clinical settings, most notably hyperthermia as adjuvant treatment with chemo- or radiotherapy in some cancers (reviewed in (79) and hypothermia for neuroprotection (reviewed in (80)). Despite the clinical use, the molecular mechanisms of thermotherapies are not well understood. A better molecular understanding of the impact of temperature on mammalian/human cells and the mechanism of temperature sensing will enable a better-targeted use of thermotherapies and may lead to new therapeutic approaches.

To further study and identify temperature sensors in humans, *in vivo* structure probing approaches, combined with RNA sequencing, Ribosome profiling and proteomics at various temperatures will be required. Additionally, the role of nucleic acid modifications in temperature sensing in humans is poorly understood. Advances in detecting (low-abundance) modifications, for example, with nanopore sequencing will aid in understanding their potential role in temperature regulation. For known temperature sensors, such as TRPV1, additional structural information at different temperatures will be crucial to understanding the molecular mechanism of their activation. To date, all full-length TRVP1 structures that show a heat-induced opening, are additionally bound by capsaicin. A potential explanation for the requirement of capsaicin in these structures is that the TRVP1 channels are more difficult to open in the nanodiscs used for these experiments compared to the endogenous cell membrane. Advances in cryo-electron tomography could circumvent the usage of nanodiscs, and allow structure determination in cellular environments.

## Data availability

All supporting data are provided within the manuscript, supplementary data and supplementary tables.

## Conflict of interest

The authors declare that they have no conflicts of interest with the contents of this article.
